# Predictive value of serum inflammatory biomarkers in postmenopausal osteoporosis: A cross-sectional study in northwest Iran

**DOI:** 10.1016/j.heliyon.2024.e36247

**Published:** 2024-08-13

**Authors:** Somayyeh Sarrafi, Leila Vahedi, Samira Pourzainali, Minoo Ranjbar, Azizeh Farshbaf-Khalili, Soraya Babaie

**Affiliations:** a Department of Midwifery, Bonab Branch, Islamic Azad University, Bonab, Iran; b Road Traffic Injury Research Center, Tabriz University of Medical Sciences, Tabriz, Iran; c Amiralmomenin Hospital of Charoimagh, Vice Chancellor for Treatment, Tabriz University of Medical Sciences, Tabriz, Iran; d Department of Midwifery, Faculty of Nursing and Midwifery, Tabriz University of Medical Science, Islamic Azad University, Tabriz, Iran; e Physical Medicine and Rehabilitation Research Centre, Aging Research Institute, Tabriz University of Medical Sciences, Tabriz, Iran

**Keywords:** Osteoporosis, Postmenopausal women, Inflammatory biomarkers, Interleukin-6, Tumor necrosis factor α

## Abstract

The purpose of this study was to compare the inflammatory biomarkers in postmenopausal women with osteoporosis and those with normal bone mineral density (BMD).

A total of 850 postmenopausal women aged 50 to 65 were randomly selected for participation in this cross-sectional investigation. 100 women displayed normal BMD, while 101 were diagnosed with osteoporosis, as determined by dual-energy X-ray absorptiometry. Biochemical techniques were used to quantify tumor necrosis factor α (TNF-α) levels, high-sensitivity C-reactive protein (hs-CRP), and interleukin-6. The area under the curve (AUC) for the diagnosis of osteoporosis was calculated using receiver-operator characteristic (ROC) curves.

A significant difference was observed between the two groups in terms of age, menopause age, education level, and BMI (p < 0.005). Moreover, TNF-α (*p* = 0.026) and hs-CRP (*p* < 0.001) levels were significant differences between two groups. The logistic regression analysis adjusted for the confounders showed that only the elevation of hs-CRP had a significant effect on the risk of osteoporosis (OR (95 % CI):42.41 (12.66–142.3), *p* < 0.001). ROC analysis demonstrated that at the cut-off point of 0.415, the sensitivity and specificity values of 83.2 % and 82.2 % were obtained, respectively, for hs-CRP.

hs-CRP is a valuable test for screening osteoporosis in postmenopausal women due to its accuracy and cost-effectiveness.

## Graphical abstract

Elevated hs-CRP serum levels increase the risk of osteoporosis in postmenopausal women.

**Figure:**
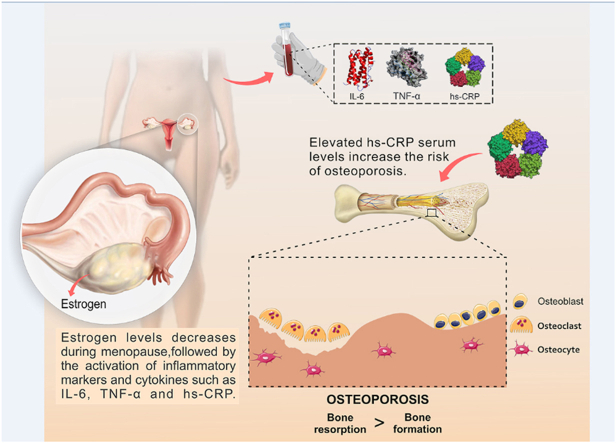

## 1 Introduction

Osteoporosis is a musculoskeletal disorder that has a profound effect on socioeconomic systems. It is characterized by diminished bone mass resulting from an imbalance between bone resorption and bone formation, ultimately leading to changes in the microarchitecture of the skeletal framework [1]. The deterioration of the microarchitecture of bones results in an increased susceptibility to fractures, thereby leading to potential functional limitations and incapacitation [2]. Osteoporosis affects about 10 % of the global population and 30 % of postmenopausal women. Its prevalence in Iran is about 32 %, almost in line with the global average. Estimations propose that about 10 million Iranian individuals suffer from this particular ailment, while approximately 34 million individuals residing in the nation exhibit lower bone mineral density (BMD) and are at an elevated risk of developing OP [3].

In contrast to numerous chronic diseases that manifest through multiple indications and symptoms, the presence of OP remains asymptomatic until the occurrence of a fracture [4]. The clinical importance of OP resides in the susceptibility to bone fractures. Based on the projections of the International Osteoporosis Association, it is anticipated that in East and Southeast Asia by the year 2050, osteoporotic fractures will account for greater than 50 % of entire broken bone [5]. The primary cause of OP in women is menopause, which is accompanied by reduced levels of sex steroid hormones [6,7].

Moreover, menopause leads to a decline in estrogen levels, triggering the activation of inflammatory cytokines like Interleukin-6 (IL-6) and Tumor necrosis factor α (TNF-α), which pave the way for the development of OP [8,9]. Cytokines play an important role in regulating bone metabolism [10]. IL-6 emerges as a potent osteoporotic cytokine with significant involvement in enhancing bone resorption and activating osteoclasts [10]. Bone repair is a process with stages of inflammation and regeneration. Interleukin-6 (IL-6) can play a major role in regulating this process. IL-6 is an immunomodulatory cytokine, transmitting IL-6 signals by two different mechanisms that include classical signaling through the membrane-bound receptor (mIL-6R) and trans-signaling through its soluble form (sIL-6R). Classical IL-6 signaling involves the binding of IL-6 to the membrane-bound IL-6 receptor α subunit and glycoprotein 130 signaling subunit. Instead, in IL-6 trans-signaling, signaling complexes IL-6 and the soluble form of the IL-6 receptor through membrane-bound glycoprotein 130. Classical IL-6 signaling can have beneficial effects in inducing a balanced immune response after fracture and bone repair after acute injury but IL-6 trans-signaling seems to have a minor role. IL-6 trans-signaling has been suggested as the predominant pathway through which IL-6 induces pathogenicity [[11], [12], [13]]. TNF-α, a cytokine primarily produced by innate immune cells like macrophages, naturally participates in inflammatory and immune responses. This mediator has the potential to worsen OP by promoting the growth and actuation of osteoclasts [14,15]. Furthermore, when exposed to substances left or unleashed by adipocytes, C-reactive protein is produced [15,16]. C-reactive protein (CRP), the production of which is stimulated by IL-6. IL-6 is a central mediator of the acute phase response and a primary determinant of hepatic production of CRP. IL-6 acts on liver cells to cause the expression of serum CRP, which is also known as acute phase reactant. CRP has two isoforms, one of which is produced locally in inflamed or damaged tissues. The other isoform is normally produced in the absence of inflammation and may have net anti-inflammatory effects [17,18]. The rapid increase in the levels of high-sensitivity C-reactive protein (hs-CRP) during the first 6–8 h after infection leads to the release of unstable molecules and enhances the activity of cells responsible for engulfing and destroying foreign particles. Consequently, this promotes the development of OP [15,16]. Inflammatory mediators contribute to a decrease in BMD by accelerating the rate at which osteocytes undergo programmed cell death. However, additional research is required to comprehensively understand the molecular and cellular mechanisms that connect inflammatory markers to the process of bone metabolism [19].

Contrary to the elevation of inflammatory moderators subsequent to menopause, limited research has been carried out in Iran to inquire about inflammatory markers in postmenopausal females with OP. Consequently, this research was carried out to compare the inflammatory agents among postmenopausal women with OP and women with normal BMD and investigate their diagnostic value in Tabriz, located in the northwest region of Iran.

## 2 Materials and methods

### 2.1 Study type and participants

The present investigation was performed as a cross-sectional and case-control descriptive research. This study obtained approval code from the Research Ethics Committee of Tabriz University of Medical Sciences, Iran (IR.TBZMED.REC.1397.733). Prior to commencing the recruitment of participants, the necessary permissions were taken from the healthcare facilities of Tabriz. The research encompassed postmenopausal individuals who possessed an electronic document in the healthcare facilities of Tabriz. Each participant signed an Informed Consent Form for the study. From a total of 850 qualified postmenopausal females, 100 individuals who had normal BMD and 101 individuals diagnosed with OP (confirmed through dual-energy X-ray absorptiometry (DEXA)) subsequent to excluding other secondary etiologies of OP were chosen as participants for the research.

The inclusion criteria included postmenopausal females who are between the ages of 50 and 65 and residing in Tabriz, who had experienced a cessation of menstruation for a minimum of 12 in succession months, without any history of abnormal fractures in the preceding decade, effective communication skills to respond to queries, onset of menopause after the 40 years old, and absence of hormone therapy in the preceding year.

Exclusion criteria encompassed the distinguish of bone diseases except OP by an endocrinologist, presence of hereditary disorders (such as hemochromatosis, hemophilia, and thalassemia), confirmation of endocrine disorders (including type 1 diabetes, hyperthyroidism, Cushing's syndrome, primary hyperparathyroidism, and renal failure and diseases) by an endocrinologist, hepatic and gastrointestinal disorders (such as chronic liver diseases like celiac disease, primary biliary cirrhosis, total or partial gastrectomy, Crohn's disease), rheumatoid arthritis, taking of any medications that influence bone metabolism (like intravenous bisphosphonate) within the past five years, taking bisphosphonate orally within the past six months, the collective utilization of oral bisphosphonate for a duration surpassing three years or longer than 30 days in the previous 6–12 months before the commencement of the investigation, administration of parathyroid hormone analogues or Strontium during the previous year, excessive intake of immunosuppressive drugs (like cyclosporines), cytotoxic, thyroxine drugs, the utilization of corticosteroids or hormonal medications throughout the duration of the investigation, prolonged use of certain anticonvulsants (like phenytoin), hypocalcemia, D25 (OH) concentration below 20 ng/mL (indicative of secondary OP), and lastly, a body mass index (BMI) below 18.5 [20]. The eligibility criteria and distinctness between primary and secondary OP were evaluated by a teammate who was an endocrinologist. Osteoporosis was identified as reduced BMD in the femoral neck or lumbar vertebrae when the T-score reached a value of −2.5 or below; osteopenia was classified as a T-score ranging from −1 to −2.5, and a T-score equal to or exceeding −1 was designated as normal.The city of Tabriz encompasses a total of 87 health centers, which provide comprehensive details and contact information for all postmenopausal women. By utilizing the integrated health system, we first compiled a comprehensive record comprising 50-65-year-old women at the postmenopausal stage enrolled across all 87 healthcare centers. Out of a total population of 108,778 individuals, a sample of 850 women (approximately double the needed sample quantity or amount) was selected using simple probability sampling. Subsequently, the selected individuals were contacted via telephone and given a short explanation of the research's aims and protocols. Upon their agreement to participate, they were requested to visit the healthcare facilities at a designated time. In total, 730 eligible individuals were interviewed through telephone conversations and instructed to attend the healthcare facilities on particular dates. During face-to-face meetings, the study's aims were thoroughly clarified, and the form of inclusion and exclusion criteria was meticulously completed. At this stage, 194 individuals who did not meet the inclusion and exclusion criteria “according to study checklist” were omitted from the research.

Ten milliliters of blood samples were taken from the existing 536 individuals in order to conduct an analysis of complete blood count with differential and determine the concentrations of thyroid stimulating hormone, calcium, vitamin D, fasting blood sugar, Alkaline phosphatase, phosphorus, and creatinine to distinguish between primary and secondary OP. These blood samples were subjected to analysis in the lab of the Nutrition Research Center at Tabriz University of Medical Sciences, under the guidance of an expert. The final diagnosis of OP, as well as the differentiation between primary and secondary OP, was made by the endocrinologist of our research team. Based on the findings of these tests, 74 individuals were distinguished with secondary OP and subsequently excluded from the investigation. Additionally, 17 women who demonstrated an unwillingness to keep participating in the research were also excluded. In the end, midwifery and demographic questionnaires were filled out for a total of 445 participants, who were subsequently referred to Sina Hospital of Tabriz in order to determine the BMD of the femoral neck and lumbar vertebrae using DEXA. All measurements were carried out by an identical expert. In order to measure BMD, the standards of T score (which signifies the quantity of SD or standard error among the sufferer's - BMD and the mean - BMD in youthful individuals) and Z score (which signifies the quantity of SD betwixt or among the sufferer's BMD and the mean BMD for individuals of the identical age and weight) were established, and BMD was ascertained in units of g/cm2.

As stated by the guidelines provided by the World Health Organization (WHO), T scores that are equal to or greater than −1, between −1 and −2.5, and less than or equal to −2.5 are categorized as normal, osteopenia, and osteoporosis, respectively.

These classifications are based on the BMD falling below 2.5 SD of that which is observed in a 30-year-old male or female [21]. Densitometry analysis reports revealed that out of the total individuals, 142 had normal BMD, 109 participants were diagnosed with osteoporosis, and 194 individuals had osteopenia. For further investigation, a subgroup consisting of 100 individuals with normal BMD and 101 individuals with OP were selected randomly based on sample size equation. In order to measure the concentrations of inflammatory markers such as and IL-6, TNF-α, hs-CRP, blood samples were collected from these individuals while they were fasting. The collection of blood samples was conducted utilizing tubes containing serum separator gels. Subsequently, these samples were centrifuged and then stored at a temperature of −80 °C. The measurements and conducting biochemical assays were dutifully carried out by researchers affiliated with the Nutrition Research Center, under the careful guidance of a laboratory expert.

### 2.2 The sample size

Sample size was determined using the G-Power software ver. 3.1.2 (Heinrich-Heine-Universität Düsseldorf, Düsseldorf, Germany; http://www.gpower.hhu.de/) based on the study by Zheng et al. [22] which reported that the mean scores and standard deviation (SD) of IL-6 and TNF-a in healthy women were 14700 ± 4810, and 12340 ± 5250 respectively, and in women with osteoporosis were 21920 ± 8310, and 15900 ± 6560 respectively. Accordingly, with a power of 0.95 and α and β value as 0.5, sample size per group was determined to be 24 for the IL-6 variable, and 74 for the TNF-a variable. Considering a dropout rate of 30 %, the final sample size was increased to 100 for each group.

### 2.3 The evaluation of study variables

Measurement of study variables involved the use of a demographic questionnaire to gather information on age, occupation, menopausal age, marital status, education level, vacancy status, adequacy of monthly income, hookah use, smoking, alcohol consumption, sunlight exposure, physical activity, family history of bone fractures due to OP, supplementation use, history of hypertension and dyslipidemia, and BMI.

The assessment of physical activity level took place using a validated and reliable short version of the International Physical Activity Questionnaire (IPAQ) [23]. This questionnaire was administered through interviews, whereby the degree of physical activity was categorized as either low, medium, or high.

The dietary consumption was evaluated through a three-day food enrolment question sheet or survey. This questionnaire encompassed two inconsequent working days or work days and one day of the weekend.

The data obtained on the aggregate energy, macronutrients, fiber, minerals, and vitamins were scrutinized through the utilization of Nutritionist IV software, which had been adjusted to accommodate Iranian cuisine.

The serum concentrations of TNF-α and IL-6 were determined via the utilization of trading kits procured by Carmania Pars Gene Co., adhering to the provided instructions. The samples were examined at 450 nm applying an ELISA reader, and the optical density measurements were employed to ascertain the serum concentration of the cytokines. The hs-CRP serum concentration was quantified by a trading kit from Pars Azmoun Co. with the aid of an auto-analyzer. This kit used an enhanced turbidimetric technique that facilitated complex formation between hs-CRP and antiserum.

To guarantee the scientific validity of the instruments employed in this investigation, the logical validity technique was employed. The reliability of the tests was evaluated by analyzing the initial 10 samples twice under different names, and the reliability of the test outcomes was computed [24].

### 2.4 Statistical analysis

The evaluation of the normality of numerical variables was performed utilizing the Kolmogorov-Smirnov test, which indicated the absence of a Gaussian distribution of inflammatory biomarkers among the investigation groups. Descriptive statistics, encompassing percentage, frequency, SD, and mean, were employed to present the midwifery and socio-demographic characteristics. The study variables were contrasted between the two investigation groups (i.e., women afflicted with OP and women with normal BMD) applying the independent *t*-test (for data that adhered to a normal distribution) or the Mann-Whitney test (for data that did not adhere to a normal distribution). The determination of correlations between BMD in the femoral neck and lumbar spine was accomplished via the Spearman correlation test. In addition, a logistic regression method was employed to adjust for the influence of confounders (age, menopause age, BMI, and education), whereby the probability of OP is computed for each investigation factor. The adequacy of the technique was assessed using the Hosmer and Lemeshow test. To explore the ability of hs-CRP to differentiate osteoporosis in postmenopausal women aged 50 to 65, we utilized receiver operating characteristic (ROC) analysis. The diagnostic accuracy was assessed using the Area Under Curve (AUC) classification, with values ranging from 0.6 to 0.7 classified as poor, 0.7 to 0.8 as fair, 0.8 to 0.9 as good, and 0.9 to 1.0 as excellent [21]. Data analysis was performed using SPSS version 23 (SPSS, Chicago, IL, USA). A p value less than 0.05 was considered statistically significant.

## 3 Results

**Fig. 1:**
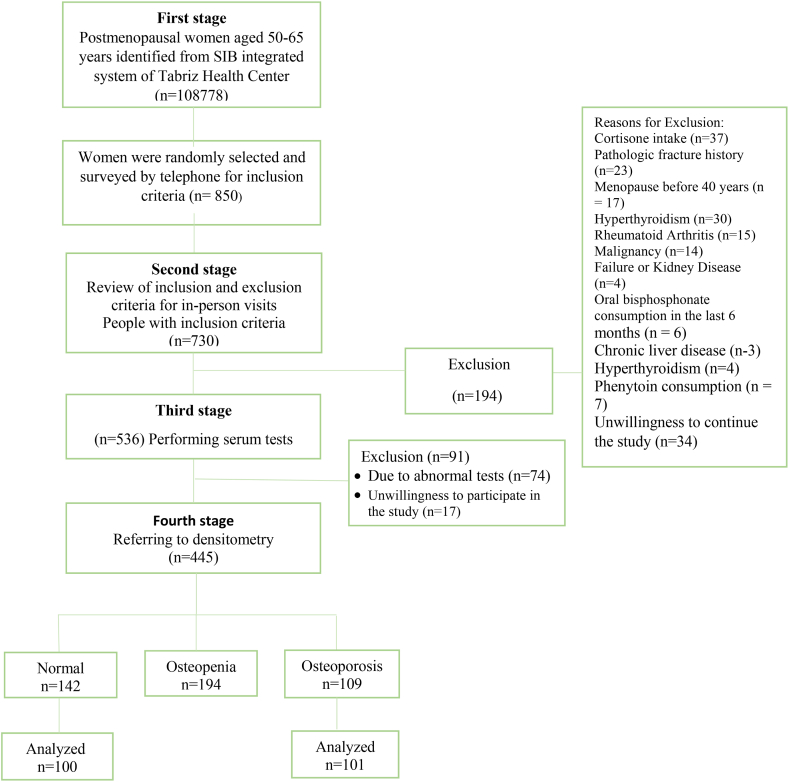
The process of selecting study participants.

In this investigation, the serum inflammatory markers were compared between 100 females who had normal BMD and 101 females who had afflicted with OP. The selection of participants is presented in Fig. 1, and Table 1 provides a comprehensive depiction of the demographic characteristics of the participants as well as a comparison of their daily food intake among the two study groups. The results of this research revealed statistically significant disparities - among the two research groups concerning age (p < 0.001), BMI (p < 0.001), age at menopause (p = 0.034), and level of education (p = 0.002). Specifically, the average age of osteoporotic women was greater; whereas the average BMI, level of education, and age at menopause were less when compared to their counterparts with normal BMD. There were no significant distinctions between the two investigation groups concerning other demographic attributes. Moreover, the mean values of T-score, Z-score, and BMD in the lumbar spine and femoral neck were significantly diminished in the osteoporotic women compared to the normal BMD group.

**Table 1:** Socio-demographic characteristics of postmenopausal women by bone density.

Variable	Normal-BMD (n = 100) n (%)	Osteoporosis (n = 101) n (%)	P
**Age** Mean (SD)	55.3 (3.6)	58.3 (3.7)	**<0.001**^c^
**Menopausal age** (year) Mean (SD)	49.3 (3.5)	48.2 (3.9)	**0.034**^c^
**Job**			0.140^a^
Housewife	84 (84.0 %)	92 (91.1 %)	
Employed	16 (16.0 %)	9 (8.9 %)	
**Adequacy of family income**			0.394^b^
Adequate	24 (24.0 %)	18 (17.8 %)	
Somewhat adequate	66 (66.0 %)	68 (67.3 %)	
Insufficient	10 (10.0 %)	15 (14.9 %)	
**Marital status**			0.079^a^
Single, divorced, widow	15 (15.0 %)	26 (25.7 %)	
Married	85 (85.0 %)	75 (74.3 %)	
**Education**			**0.002**^b^
Illiterate	15 (15.0 %)	33 (32.7 %)	
Primary school	31 (31.0 %)	40 (39.6 %)	
Secondary school	15 (15.0 %)	11 (10.9 %)	
High school/Diploma	27 (27.0 %)	12 (11.9 %)	
Academic	12 (12.0 %)	5 (5.0 %)	
**Smoking status**			0.683^a^
No smoking or history of use	98 (98.0 %)	97 (96.0 %)	
Current use or previous use	2 (2.0 %)	4 (4.0 %)	
**Exposure to direct sunlight** (Yes)	42 (42.0 %)	45 (44.6 %)	0.776^a^
Number of exposed days/week Mean (SD)	4.4 (2.3)	4.2 (2.3)	0.687^a^
Number of exposed minutes/day Mean (SD)	33.2 (21.8)	33.0 (23.5)	0.957^a^
**Supplement use**: Ca, Vit D (Yes)	50 (50.0 %)	59 (58.4 %)	0.259^a^
**Mild physical activity** (Yes)	100 (100.0 %)	100 (99.0 %)	1.0^a^
**Moderate physical activity** (Yes)	23 (23.0 %)	29 (28.7 %)	0.421^a^
**Vigorous physical activity** (Yes)	14 (14.0 %)	15 (14.9 %)	1.0^a^
**Fracture history in close relatives** (Yes**)**	8 (8.0 %)	17 (16.8 %)	0.086^a^
**Body Mass Index** (kg/m2) Mean (SD)	31.3 (5.0)	28.4 (3.7)	**<0.001**^c^
**Hypertension** (Yes)	39 (39.0 %)	39 (38.6 %)	1.0^a^
**Dyslipidemia** (Yes)	8 (8.0 %)	10 (9.9 %)	0.806^a^
**BMD/L.S** (g/cm2) Mean (SD)	1.0 (0.2)	0.71 (0.07)	**<0.001**^c^
**T-score/L.S** Mean (SD)	−0.11 (0.9)	−3.09 (0.6)	**<0.001**^c^
**Z-score/L.S** Mean (SD)	0.97 (0.9)	−1.7 (0.7)	**<0.001**^c^
**BMD/F.N** (g/cm2) Mean (SD)	0.97 (0.1)	0.75 (0.1)	**<0.001**^c^
**T-score/F.N** Mean (SD)	0.18 (0.8)	−1.6 (0.7)	**<0.001**^c^
**Z-score/F.N** Mean (SD) **Fat(g/d)** Mean (SD) **Energy(Kcal/d)** Mean (SD) **Carbohydrate(g/d)** Mean (SD) **Protein(g/d)** Mean (SD) **Vitamin D(μg/d)** Mean (SD) **Vitamin C(mg/d)** Mean (SD) **Zinc(mg/d)** Mean (SD) **Selenium(μg/d)** Mean (SD)	0.92 (0.8) 68.61(19.1) 1905.3(419.4) 272.7(70.7) 58.8(17.2) 1.8 (1.2) 226.2(119.7) 8.5(2.4) 87.0(33.8)	−0.69 (0.8) 71.62(26.43) 1818.2(658.4) 255.5(108.1) 51.6(18.0) 1.6(1.3) 203.6(126.8) 8.3(3.0) 84.0(32.1)	**<0.001**^c^ **0.478**^c^ **0.392**^c^ **0.312**^c^ **0.029**^c^ **0.363**^c^ **0.222**^c^ **0.220**^c^ **0.350**^c^

BMD: Bone mineral density; L.S: Lumbar spine; F.N: Femoral neck.

P<0.05 has been considered as a significant level.**p-value related to Fat, Energy, Carbohydrate, Vitamin D, Vitamin C, Zinc, Selenium should be unbolded.**

a Fisher's exact test.

b Linear by linear chi-square.

c Independent t-test.

Table 2 demonstrates the serum concentration of IL-6, as indicated by the median (IQR), which was 19.26 (6.26) pg/mL in the normal BMD group and 19.42 (7.75) pg/mL in the group with OP. Based on the findings of the Mann-Whitney test, the difference in IL-6 levels among both groups did not yield any statistically meaningful results (P = 0.289). In terms of TNF-α levels, the median (IQR) was 20.76 (16.84) pg/mL in the OP group, which was notably less than the median (IQR) of 25.45 (13.03) pg/mL seen in the normal BMD group (P = 0.026). Furthermore, upon evaluating hs-CRP levels, it was determined that the medians (IQRs) were 0.31 (0.67) mg/L and 0.95 (0.67) mg/L in the normal BMD and OP groups, respectively, thereby revealing a noteworthy greater concentration in the latter group (p < 0.001).

**Table 2:** Serum inflammatory biomarkers among postmenopausal women with normal bone density and osteoporosis.

Inflammatory biomarkers	Normal-BMD (n = 100)		Osteoporosis (n = 101)		p*
	Mean (SD)	Median (IQR)	Mean (SD)	Median (IQR)	
**Interleukin-6** (pg/ml)	22.14 (17.03)	19.26 (6.26)	26.41 (26.76)	19.42 (7.75)	0.289
**Tumor necrosis factors** (pg/ml)	33.76 (27.29)	25.45 (13.03)	33.60 (31.39)	20.76 (16.84)	**0.026**
**hs-CRP** (mg/L)	0.28 (0.33)	0.25 (0.31)	0.96 (0.58)	0.95 (0.67)	**<0.001**

BMD: bone mineral density, p* Mann-Whitney U.

**Fig. 2:**
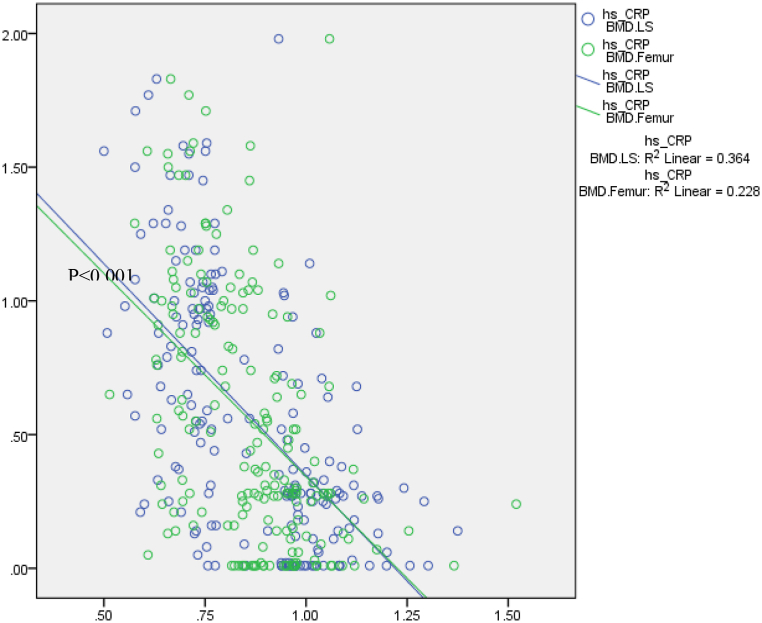
Scatter plot of correlation between hs-CRP (mg/L) with BMD (g/cm^2^) of lumbar spine (r = −0.60) and femoral neck (r = −0.51).

A moderate correlation was discovered between BMD and hs-CRP of both the femoral neck (r = −0.51, p < 0.001) and the lumbar spine (r = −0.60, <0.001) (Fig. 2).

Table 3 presents the findings of the binary logistic regression technique, which aimed to predict the likelihood of OP in females at the menopausal phase. This prediction was made by evaluating inflammatory biomarkers while controlling for age, age at menopause, level of education, and BMI. The findings indicate that there is no significant correlation between the odds of developing OP and the serum concentrations of TNF-α (p = 0.391) and IL-6 (p = 0.628). Nonetheless, there was a notable increase in the risk of osteoporosis when the hs-CRP levels were elevated (OR (95 % CI) = 42.41 (12.66–142.3), p < 0.001).

**Table 3:** Binary logistic regression model for estimating the odds of primary osteoporosis based on serum inflammatory biomarkers in postmenopausal women.

Inflammatory biomarkers	B	Adjusted Odds ratio	95 % confidence interval	P^a^
**Interleukin-6** (pg/ml)	0.004	1.004	0.987 to 1.02	0.628
**Tumor necrosis factor** (pg/ml)	−0.005	0.995	0.984 to 1.007	0.391
**hs-CRP** (mg/L)	3.747	42.41	12.66 to 142.03	**<0.001**

Interleukin-6: Hosmer and Lemeshow p = 0.182, Chi-square = 11.36 ، df = 8.

Tumor necrosis factors: Hosmer and Lemeshow p = 0.391 ،Chi-square = 8.45 ، df = 8.

hs-CRP: Hosmer and Lemeshow p = 0.551, Chi-square = 6.86, df = 8.

a Adjusted for age, menopause age, BMI, and education. Reference group: Normal-BMD.

### 3.1 Diagnostic accuracy of hs-CRP

**Fig. 3:**
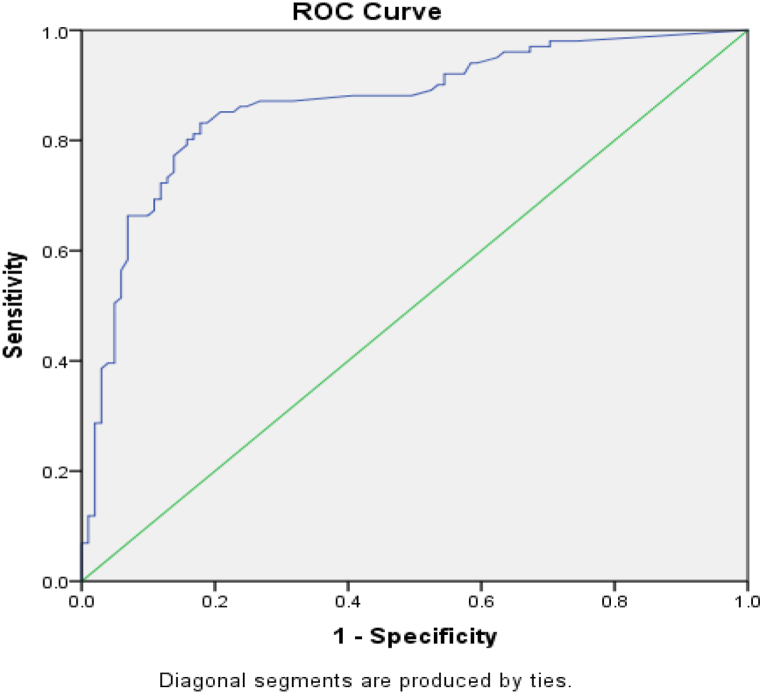
Area under the Receiver-Operator Characteristic (ROC) curve for measurement of hs-CRP in postmenopausal women with osteoporosis.

To assess the diagnostic accuracy of hs-CRP, TNF-α, and IL-6 compared to the DXA standard method for osteoporosis, we employed the ROC curve (Fig. 3). The AUC, along with its 95 % confidence interval, standard error, and p-value for hs-CRP were obtained as follows: 0.87 (95 % CI: 0.81 to 0.92, SE: 0.026, p < 0.001). The cut-off point of 0.415 yielded a sensitivity of 83.2 %, specificity of 82.2 %, positive likelihood ratio of 4.67, negative likelihood ratio of 0.20, positive predictive value of 82.3 %, and negative predictive value of 83 %. The AUC with 95 % confidence interval, standard error, and p-value for TNF-α, and IL-6 were 0.40 (95 % CI: 0.32 to 0.48, SE: 0.041, p = 0.018) and 0.54 (95 % CI: 0.46 to 0.62, SE: 0.041, p = 0.331), respectively.

## 4 Discussion

The discoveries of this research demonstrated that females afflicted with OP exhibited a greater average age while a less average age at menopause, level of education, and BMD compared to women with a normal BMD. Whereas there was a marked disparity among the two investigation groups regarding the hs-CRP and TNF-α concentrations, the risk of OP only escalated significantly in association with increasing hs-CRP levels after controlling for confounding factors. In accordance with the findings, each unit (1 mg/L) increment in the hs-CRP concentrations – increased the chance of OP greater than 40 times. Hs-CRP indicated good diagnostic value for OP in the early postmenopausal period.

During the menopausal phase, inflammatory cytokines like TNF-α and IL-6 are activated alongside the decline in estrogen concentration, which act as an antioxidant agent that enhances total antioxidant capacity (TAC). This activation contributes to the development of osteoporosis [8]. One During menopause, the activity of bone-resorbing cells increases due to declining estrogen levels, leading to a 50 % reduction in spongy bone mass and a 30 % decrease in dense bone mass, resulting in a higher risk of bone fractures in individuals aged 60 to 75 [25]. In estrogen deficiency, the activity of osteoclasts becomes heightened, resulting in an escalation of bone loss and a reduction in bone density. Consequently, the rate of bone loss in postmenopausal females experiences an annual increase of 2–3% owing to the diminished levels of estrogen [26].

Consistent with our findings, Benjamas et al. conducted a cross-sectional examination including 102 men with chronic obstructive pulmonary disease (COPD) and found that BMI and hs-CRP were significantly associated with OP. Furthermore, the recent investigation also revealed that patients afflicted with OP exhibited lower BMIs and higher hs-CRP levels in comparison to those with normal BMD [26]. Similarly, Sudjaroen et al. found through a cross-sectional analysis involving 105 adults aged over 60 years old that the concentration of hs-CRP was higher in individuals with OP compared to those with normal BMD, which is consistent with the results of our study [27]. A study in Australia demonstrated that increased hs-CRP concentrations were linked to a higher risk of fracture, but variations in methodologies and sample sizes should be considered [28]. However, it is essential to acknowledge that there existed several dissimilarities between the recent study and our own research, encompassing variations in the methodologies employed to measure inflammatory biomarkers, inclusion criteria, sample size, and follow-up duration. In a case-control investigation conducted in Iran in 2019, Safari et al. examined the correlation between circulating vitamin D, amyloid A, and hs-CRP levels with OP in women at the menopausal stage. The study compared 44 postmenopausal women diagnosed with OP, referred to as the case group, to 44 postmenopausal women without OP, referred to as the control group. The study yielded no statistically significant distinction in hs-CRP concentrations among the two groups [29]. These findings contradict our own observations, which could potentially be attributed to disparities in sample size, the age range of participants, as well as the exclusion of secondary etiologies of OP in our research. Similarly, a study on mice showed that estrogen deficiency correlated with decreased bone density due to the regulation of TNF-α and IL-6. However, the correlation between TNF-α and OP disappeared after adjusting for confounding factors. Another study revealed elevated TNF-α levels in postmenopausal women with OP [30].

In a study conducted in the year 2018, Lizha et al. demonstrated that TNF-α could potentially have a role in postmenopausal afflicted with OP by enhancing the process of RANKL-stimulated osteoclastogenesis. TNF-α levels were found to be markedly elevated in postmenopausal women with OP when compared to those without this condition [31]. It is important to note that in our investigation, we specifically excluded secondary causes of OP, a step that was not taken in Lizha et al.'s study. In addition, our study had a smaller sample size compared to theirs. Zheng et al. showed that direct stimulation resulted in increased concentrations of IL-6, IL-1β, TNF-α, IFN-γ, LIF, and GM-CSF. Among these, the production of TNF-α and IL-6 was significantly higher in women with OP compared to healthy individuals in the control group [22].

The disparity between our results and those of Zheng et al. may be attributed to disparities in inclusion criteria and sample size. In Saudi Arabian research conducted in 2017, Al-Daghri et al. sought to examine the correlation between bone turnover factors and pro-inflammatory agents in postmenopausal females with and without OP. The findings of the aforementioned investigation demonstrated that TNF-α and IL-6 levels were notably elevated in women afflicted with OP in comparison to healthy women [32]. The disparity between our findings and those of the last research could be or may be explicated by the divergent age ranges of the individuals in both types of research (50–65 years old versus – greater than 50 years old). Furthermore, both investigations employed distinct methodologies to evaluate the inflammatory biomarkers. To mitigate selection bias, the random sampling approach was implemented. Participants were chosen from postmenopausal women at healthcare facilities in varying socio-economic areas in Tabriz city to share findings with a wider community. Women with secondary OP and certain lifestyle were excluded. Women aged 50–65 were selected due to BMD decline after decreased estrogen levels. Findings may not apply to postmenopausal women over 65. Results are not generalizable to the entire population, including men. However, a primary limitation of current investigation was the restricted examination of various inflammatory factors. Thus, it is recommended that future investigations be conducted to screen other inflammatory markers such as IL-1 ‘which have an impact on osteoclastogenesis and bone resorption’ and combine them with CRP to identify if they offer better predictive metrics. Moreover, we suggest to screen for a panel of inflammatory markers and then down select among them to accurate prediction of postmenopausal osteoporosis.

Moreover, in the current investigation the studied biomarkers were used as an early diagnosis of osteoporosis. It is suggested to conduct cohort studies, in which high levels of inflammatory biomarkers are analyzed to assess the risk of developing osteoporosis. Identification can be a valuable tool for prognostic tests.

## 5 Conclusion

Considering the increasing significance of OP in the country's healthcare system, especially in light of changing demographic trends, as well as the high prevalence and debilitating effects of this disease, it is imperative to investigate the role of inflammatory biomarkers and their upward trajectory. Hs-CRP indicated a good predictive value for postmenopausal OP in current study. The cost-effectiveness and accessibility of measuring hs-CRP make it a valuable tool for the prevention and early detection of OP in women at the postmenopausal stage.

## Funding

The project has been funded by the Research and Technology Deputy of Tabriz University of Medical Sciences (grant no: 64575).

## Ethics approval

This study obtained approval code from the Research Ethics Committee of Tabriz University of Medical Sciences, Iran (IR.TBZMED.REC.1397.733).

## Data availability statement

The data that support the findings of this study are available from the corresponding author upon reasonable request.

## CRediT authorship contribution statement

**Somayyeh Sarrafi:** Writing – original draft, Methodology. **Leila Vahedi:** Validation, Data curation. **Samira Pourzainali:** Writing – original draft. **Minoo Ranjbar:** Writing – original draft. **Azizeh Farshbaf-Khalili:** Writing – original draft, Validation, Supervision, Formal analysis. **Soraya Babaie:** Supervision, Project administration, Data curation.

## Declaration of competing interest

The authors declare that they have no known competing financial interests or personal relationships that could have appeared to influence the work reported in this paper.
